# Bone marrow concentrate promotes bone regeneration with a suboptimal-dose of rhBMP-2

**DOI:** 10.1371/journal.pone.0191099

**Published:** 2018-01-18

**Authors:** Kazuhiro Egashira, Yoshinori Sumita, Weijian Zhong, Takashi I, Seigo Ohba, Kazuhiro Nagai, Izumi Asahina

**Affiliations:** 1 Department of Regenerative Oral Surgery, Unit of Translational Medicine, Nagasaki University Graduate School of Biomedical Sciences, Nagasaki, Japan; 2 Basic and Translational Research Center for Hard Tissue Disease, Unit of Translational Medicine, Nagasaki University Graduate School of Biomedical Sciences, Nagasaki, Japan; 3 Department of Oral and Maxillofacial Surgery, College of Stomatology, Dalian Medical University, Dalian, Liaoning, China; 4 Transfusion and Cell Therapy Unit, Nagasaki University Hospital, Nagasaki, Japan; Medical University of South Carolina, UNITED STATES

## Abstract

Bone marrow concentrate (BMC), which is enriched in mononuclear cells (MNCs) and platelets, has recently attracted the attention of clinicians as a new optional means for bone engineering. We previously reported that the osteoinductive effect of bone morphogenetic protein-2 (BMP-2) could be enhanced synergistically by co-transplantation of peripheral blood (PB)-derived platelet-rich plasma (PRP). This study aims to investigate whether BMC can effectively promote bone formation induced by low-dose BMP-2, thereby reducing the undesirable side-effects of BMP-2, compared to PRP. Human BMC was obtained from bone marrow aspirates using an automated blood separator. The BMC was then seeded onto β-TCP granules pre-adsorbed with a suboptimal-dose (minimum concentration to induce bone formation at 2 weeks in mice) of recombinant human (rh) BMP-2. These specimens were transplanted subcutaneously to the dorsal skin of immunodeficient-mice and the induction of ectopic bone formation was assessed 2 and 4 weeks post-transplantation. Transplantations of five other groups [PB, PRP, platelet-poor plasma (PPP), bone marrow aspirate (BM), and BM-PPP] were employed as experimental controls. Then, to clarify the effects on vertical bone augmentation, specimens from the six groups were transplanted for on-lay placement on the craniums of mice. The results indicated that BMC, which contained an approximately 2.5-fold increase in the number of MNCs compared to PRP, could accelerate ectopic bone formation until 2 weeks post-transplantation. On the cranium, the BMC group promoted bone augmentation with a suboptimal-dose of rhBMP-2 compared to other groups. Particularly in the BMC specimens harvested at 4 weeks, we observed newly formed bone surrounding the TCP granules at sites far from the calvarial bone. In conclusion, the addition of BMC could reduce the amount of rhBMP-2 by one-half via its synergistic effect on early-phase osteoinduction. We propose here that BMC transplantation facilitates the clinical use of rhBMP-2 as an alternative strategy for bone engineering.

## Introduction

Current surgical strategies for healing bone defects arising from trauma or disease employ either autogenous bone grafts or alloplastic bone substitutes [1]. While autograft procedures can involve donor site morbidity, this strategy can be a realistic option for patients with severe bone defects [2,3]. Meanwhile, because alloplastic materials lack osteogenic potential, their application remains limited, and the results of these strategies have been inconsistent to date [4]. Therefore, tissue engineering has recently been considered a potential alternative strategy to bone reconstruction, since it is thought to be less invasive and safer than conventional methods [5,6]. For this reason, growth factors and stem cells are now receiving significant attention as key elements in tissue engineering that can confer osteoinducibility to alloplastic bone substitutes.

Recombinant human (rh) BMP-2 is one of several bone morphogenetic proteins (BMPs) and has been shown to induce bone formation in a variety of indications. Many animal studies have demonstrated the successful use of this protein for bone tissue engineering [7,8,9], and some clinical reports have demonstrated the effectiveness of direct implantation of rhBMP-2 into the body, such as in sinus augmentation or bone regeneration of the premaxillary cleft [10,11]. However, implantation of high doses of rhBMP-2 induces substantial swelling that may obstruct the airways when applied to oral and cervical areas [12]. Therefore, to expand the clinical utility of rhBMP2, recent studies have focused on reducing the dose of rhBMP-2 without attenuating its osteoinductive function by combining rhBMP-2 with other growth factors, stem cells, or sustained-release materials [13,14]. It was recently shown that co-delivery of stromal cell derived factor-1 (rhSDF-1) with a suboptimal dose of rhBMP-2 promotes osteoinduction to a level comparable to an optimal dose of rhBMP2 without apparent adverse effects in rat calvarial defects [15]. We also demonstrated that peripheral blood platelet-rich plasma (PRP) and rhBMP2 synergistically exert highly osteoinductive properties to alloplastic substitutes in rabbit calvaria, suggesting that rhBMP-2 exerts effects on osteoprogenitor cells recruited by stimulation of growth factors contained in PRP [16]. However, an efficient delivery method that avoids the undesirable side effects of rhBMP-2 remains to be established.

There are an increasing number of clinical trials using bone marrow concentrate (BMC) transplantation for treating bone or cartilage injuries such as atrophic non-union, osteoarthritis or degenerative disc disease [17–20]. BMC is also known as bone marrow aspirate-derived PRP, and these trials have focused on the effectiveness of cell therapy based not only on platelets releasing the activated growth factors but also on the population of mononuclear cells (MNCs) [18–21]. This population contains mesenchymal stem/progenitor cells (MSCs) or endothelial progenitor cells (EPCs), although the ratio of these stem/progenitor cells is extremely low [22,23]. MSCs can differentiate into multiple cell types, including osteogenic lineages, as well as display paracrine functions such as promoting angiogenesis and activating stem cells at injured sites [24]. EPCs can give rise to endothelial cells and contribute to the regeneration of damaged tissues by favoring neovascularization by direct differentiation or in a paracrine fashion at implanted sites [25,26]. Therefore, due to the different interactions of these stem/progenitor cells, including hematopoietic stem cells, cell therapy utilizing bone marrow MNCs has been considered as a potent strategy to treat tissue defects. Indeed, several studies on bone regeneration have demonstrated that these stem/progenitor cells promote osteogenesis by their synergistic effects [27,28]. In this way, MNCs in BMC may interact with rhBMP-2 at sites of bone injury and induce bone formation effectively with a reduced dose of rhBMP-2. However, further synergistic effects of BMC and rhBMP-2 have not been well examined to date.

The aim of this study was to investigate whether BMC can promote the bone augmentation induced by low-dose rhBMP-2. We previously showed that human BMC and PRP possess a similar ability to accelerate new bone formation in the early phase of bone regeneration when transplanted to mouse calvaria with alloplastic bone substitutes that have not adsorbed any growth factors (such as rhBMP-2) [21]. This result led us to consider that PRP is a more realistic clinical option for bone engineering compared to BMC. However, in consideration of the expected advantages of BMC, rhBMP-2 might provide conditions that permit more rapid and effective bone regeneration with MNCs and/or platelets in BMC. This study was a prerequisite step for future clinical trials aimed at facilitating the clinical application of rhBMP-2 for bone engineering.

## Materials and methods

### BMC and PRP Isolation

All experiments in this study were carried out in compliance with the Helsinki Declaration. Sample collection was approved by the Ethics Committee of Nagasaki University Graduate School of Biomedical Sciences (11032828), and written informed consent was obtained from all donors. Volunteer donors comprised five men aged 31 to 43 years with no history or evidence of genetic disease or malignancy.

**Fig 1:**
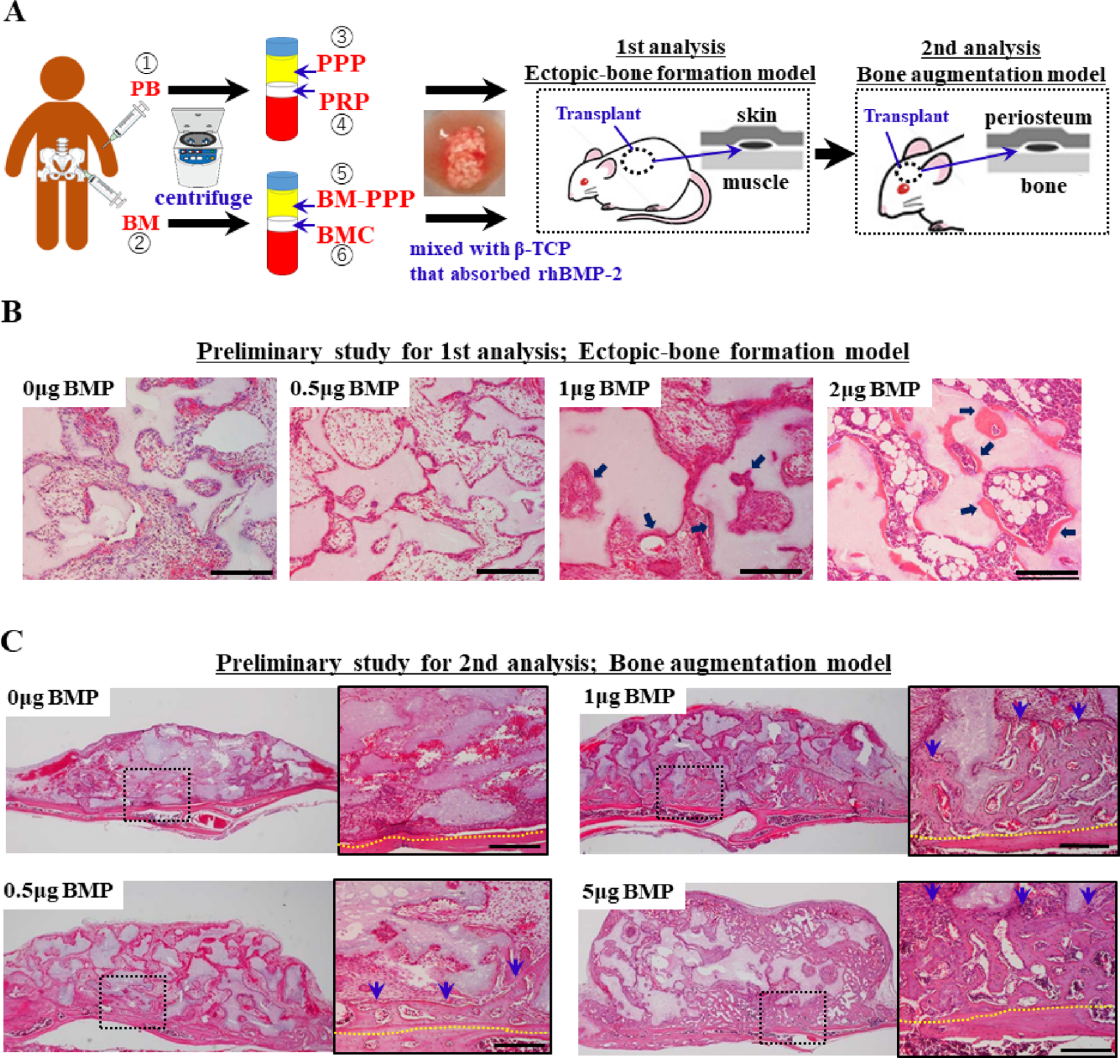
Schematic diagram of the experimental design for PB or BM-based bone engineering with rhBMP-2, and the preliminary study to determine the suboptimal-dose of rhBMP2 on bone formation. **Typical histological appearance in specimens at 2 weeks following transplantation. (A)** The 6 fractions (1, PB; 2, BM; 3, PPP; 4, PRP; 5, BM-PPP; 6, BMC) were mixed with the alloplastic material (β-TCP granules) containing a suboptimal dose (0.5 μg) of rhBMP2. **(B)** When 20mg β-TCP granules after adsorbed 0, 0,5 1 or 2 μg of rhBMP-2 were transplanted subcutaneously (as a model of ectopic bone formation), 1 μg rhBMP-2 could induce the minimal bone formation (arrows). Scale bars represent 200 μm. **(C)** When 20mg β-TCP granules after adsorbed 0, 0,5 or 1, 5 μg of rhBMP-2 were transplanted onto the cranium (as a model of vertical bone augmentation), 0.5 μg rhBMP-2 could induce the minimal bone formation. Scale bars represent 200 μm. The yellow dotted line indicates the boundary between the host bone and the specimen. Blue-arrows indicated the augmented bones.

Bone marrow (BM) was aspirated from the posterior iliac crest under local anesthesia (**Fig 1A**). Peripheral blood (PB) was obtained from the cubital vein. Equal volumes (30 ml) of BM and PB aspirate were collected with syringes containing 3 ml anticoagulant citrate dextrose solution. Next, 3 ml of plasma containing both concentrated mononuclear cells (MNCs) and platelets (PLTs) was isolated from 30 ml of each aspirate and termed as bone marrow concentrate (BMC) and platelet-rich plasma (PRP), respectively, using an automated blood cell separator (Magellan MDK 305; Ateriocyte Medical System, Cleveland, OH, USA) according to the manufacturer’s instructions. After removing BMC and PRP, 3 ml of platelet-poor plasma (PPP) fraction (BM-PPP and PPP) was collected from each supernatant. The concentrations of MNCs and PLTs in each fraction (BM, BMC, BM-PPP, PB, PRP, and PPP) were assessed before use in the *in vivo* experiments.

### Preparation of specimens for transplantation

For *in vivo* experiments, 1 μg (when transplanted subcutaneously into the mouse) or 0.5 μg (when transplanted onto the mouse cranium) of rhBMP-2 (donated by Astellas Pharma, Tokyo, Japan) was adsorbed onto 20 mg β- tricalcium phosphate (β-TCP) granules (0.5–1.5 mm size; Osferion^®^, Olympus, Tokyo, Japan), and then these materials were lyophilized. Just before transplantation, 60 μl of each fraction (BM, BMC, BM-PPP, PB, PRP, and PPP) was mixed with the lyophilized material, and then 10 μl of a bovine thrombin and 10% calcium chloride (1:1 ratio) mixture was added to the β-TCP granules/fraction mixture to trigger fibrin polymerization and produce an insoluble gel (**Fig 1A**). The final concentrations of thrombin and CaCl_2_ in the grafting aspirates were 227.3 U/ml and 4.6 mg/ml, respectively.

Regarding the rhBMP-2 dose, we performed preliminary *in vivo* experiments to determine the suboptimal-doses which can induce minimal bone formation at 2 weeks following transplantation, and determined the suboptimal-doses (as low-doses) to be 1 μg/20 mg β-TCP (when subcutaneously transplanted to the mouse) (**Fig 1B**) or 0.5 μg/20 mg β-TCP (when transplanted onto the mouse cranium) (**Fig 1C**). Each experiment was performed in triplicate for three samples.

### Transplantation

All animal experiments were performed at the Nagasaki University animal center, and all experimental procedures were performed in accordance with the protocols approved by the Animal Care and Use Committee of Nagasaki University (approved number: 0810140706).

To assess the synergistic effect of BMC and the suboptimal-dose of rhBMP-2, we first examined ectopic bone formation following their subcutaneous implantation. Then, transplantations for on-lay placement on the mouse cranium as a bone augmentation model were conducted and assessed. Surgery was performed on 140 healthy, 6-week-old female BALB/cAJcl-nu/nu mice (Nihoncrea, Tokyo, Japan) that were randomized into seven groups (**Table 1**): **1)** β-TCP transplantation without any fractions [**Control group**; n = 5 at each time point (2 and 4 weeks post-transplantation) (for subcutaneous and cranium models, respectively), Total n = 20], **2)** PB and β-TCP transplantation [**PB group**; n = 5 at each time point (2 and 4 weeks post-transplantation) (for subcutaneous and cranium models, respectively), Total n = 20], **3)** PPP and β-TCP transplantation [**PPP group**; n = 5 at each time point (2 and 4 weeks post-transplantation) (for subcutaneous and cranium models, respectively), Total n = 20], **4)** PRP and β-TCP transplantation [**PRP group**; n = 5 at each time point (2 and 4 weeks post-transplantation) (for subcutaneous and cranium models, respectively), Total n = 20], **5)** BM and β-TCP transplantation [**BM group**; n = 5 at each time point (2 and 4 weeks post-transplantation) (for subcutaneous and cranium models, respectively), Total n = 20], **6)** BM-PPP and β-TCP transplantation [**BM-PPP group**; n = 5 at each time point (2 and 4 weeks post-transplantation) (for subcutaneous and cranium models, respectively), Total n = 20], **7)** BMC and β-TCP transplantation [**BMC group**; n = 5 at each time point (2 and 4 weeks post-transplantation) (for subcutaneous and cranium models, respectively), Total n = 20]. The BM and PB groups were used as experimental controls to clarify the exact efficacy of concentrated MNCs and PLTs on the BMC and PRP groups. With regards to the two PPP groups, PPP is known to contain concentrated fibrinogen and plasmatic growth factors, and has a positive effect for osteoblastic cell differentiation [29,30]. Therefore, we considered the possibility that the PPP or BM-PPP groups would show a synergistic effect with rhBMP-2 on bone formation. The process of randomization was conducted according to 10 deliveries of 14 mice each (7 mice for each model per donor). Each group comprised 5 mice at 2 and 4 weeks post-transplantation for each model.

**Table 1:** Control- and experimental-groups for transplantation.

Group	Fraction (60μ1)	Supplementation/scaffold *^1^
Contl	non-fraction	low-dose rhBMP2 ^*2^ / β-TCP (20mg)
PB	peripheral blood	
PPP	platelet-poor plasma	
PRP	platelet-rich plasma	
BM	bone marrow aspirate	
BM-PPP	bone marrow platelet-poor plasma	
BMC	bone marrow platelet-rich plasma	

*1 bovine thrombin (10μl) and calcium chloride (10%) were added to each group before transplantation.

*2 low-dose rhBMP-2; 1.0μg for subcutaneous transplantation, and 0.5μg for calvarial transplantation.

The mice were anesthetized by intraperitoneal administration of 0.08 ml/g body weight of sodium pentobarbital, and diethyl ether was used as a supplement to maintain mild anesthesia. Then, the dorsal skin was incised and the specimens were transplanted subcutaneously (as a model of ectopic bone formation) (**Fig 1A**). Separate from the subcutaneous transplantation, the calvarial skin was incised and the periosteum reflected. The graft materials were also transplanted onto the cranial surface (as a clinical model of vertical bone augmentation) **(Fig 1A**). Vertical-type bone augmentation remains challenging clinically because of insufficient bone formation and delayed healing. Therefore, this model is useful for demonstrating the feasibility of augmenting atrophic alveolar bone with engineered bone [21,31–33]. Two and four weeks post-implantation, the mice were sacrificed by CO_2_ asphyxiation and the specimens were harvested.

### Histological and immunohistological observations

To assess new bone formation at 2 and 4 weeks following transplantation, harvested specimens were fixed with 4% paraformaldehyde, decalcified with a solution containing 2.9% citric acid, 1.8% tri-sodium citrate dehydrate, 10% formic acid, and 90% distilled water, and embedded in paraffin wax. Sections (3 μm thick) were deparaffinized and stained with hematoxylin and eosin (H&E). The volume of newly formed bone-like tissues was analyzed using ImageJ software (NIH, Bethesda, MD, USA). The percentage of surface area occupied by bone-like tissues was assessed by light microscopy under x30 magnification using five sections from each of the five specimens per group. Two examiners independently chose sections randomly in a blinded fashion and the area of new bone growth was measured.

For Masson’s trichrome staining, the slides of cranial specimens at 2 and 4 weeks post-transplantation were incubated for 15 min at 56°C in Bouin’s solution (Sigma, St. Louis, MO, USA) and then washed under running tap water to remove excess stain. The nuclei of specimens were stained with Weigert’s iron hematoxylin (Sigma) for 5 min. The slides were washed in running tap water for 5 min and rinsed in DW. The slides were placed in a phosphotungstic/phosphomolybdic acid solution for 5 min and then stained with Aniline Blue solution for 5 min. The slides were treated with 1% acetic acid for 2 min and then fixed with mounting medium (Muto Pure Chemicals, Tokyo, Japan). Five sections were stained from each of the five specimens per group.

Then, immunohistochemical staining of cranial specimens at 2 weeks post-transplantation was performed with a Vectastain ABC kit (Vector, Burlingame, CA, USA). Sections were stained with mouse monoclonal anti-human vimentin antibody (1:100; Abcam, Cambridge, UK), and the slides were incubated with anti-mouse secondary-antibody (1:200). Then, the specimens were finally reacted with 0.1% w/v 3.3’-diaminobenzidine tetrahydrochloride (DAB immunohistochemistry; GenWay Biotech, San Diego, CA, USA) in PBS and counterstained with hematoxylin. Control staining was performed by replacing the first antibody with pre-immune serum eluted from the corresponding affinity columns. Five sections were stained from each of the five specimens per group.

### Statistical analysis

All experimental values are presented as mean values ± standard deviations. Means were analyzed using one-way analysis of variance. Tukey’s multiple comparison t-test was used to detect significant differences within each group for the histological bone formation area. P < 0.05 was considered statistically significant.

## Results

### Cell numbers in each fraction

Following centrifugation, the concentration of MNCs increased by a factor of 2.4±0.4 from the BM group to the BMC group, and increased by a factor of 2.5±1.0 from the PB group to the PRP group (**Table 2**). The concentration of PLTs increased by a factor of 4.81±1.9 from the BM group to the BMC group, and increased by a factor of 4.2±2.4 from the PB group to the PRP group following centrifugation (**Table 2**). As a result, the BM and BMC groups contained approximately 2.5- and 2.6-fold MNCs and almost the same number of PLTs when compared to the PB and PRP groups, respectively. In contrast, both the BM-PPP and PPP groups contained a small number of MNCs and PLTs compared to the other groups.

**Table 2:** Numbers of mononuclear cells (MNCs) and platelets (PLTs) in each fraction.

**< MNC (x10**^**2**^**/μl) >**						
**donor/group**	**PB**	**PPP**	**PRP**	**BM**	**BM-PPP**	**BMC** (BM-PRP)
**Donor 1**	41	1	91	66	2	189
**Donor 2**	37	9	85	79	9	179
**Donor 3**	41	1	113	117	0	327
**Donor 4**	34	0	139	114	3	225
**Donor 5**	72	15	90	195	25	434
**Mean±SD**	45.0±15.4	5.2±6.6	103.6±22.5	114.2±50.3	7.8±10.2	270.8±108.4
**< PLT (x10**^**4**^**/μl) >**						
**donor/group**	**PB**	**PPP**	**PRP**	**BM**	**BM-PPP**	**BMC** (BM-PRP)
**Donor 1**	7.2	1.1	52.7	7.4	1.8	47.9
**Donor 2**	19.6	15.8	53.5	15.1	3.7	45.6
**Donor 3**	18.2	2.1	59	8.8	2.7	58
**Donor 4**	14.5	1.7	88	9.2	2.8	25.3
**Donor 5**	22.1	14.2	35.7	19.8	8.7	98.1
**Mean±SD**	16.32±5.8	6.98±7.4	57.78±19.0	12.06±5.2	3.94±2.7	54.98±26.9

### Histological observations of samples transplanted subcutaneously to the dorsal skin

**Fig 2:**
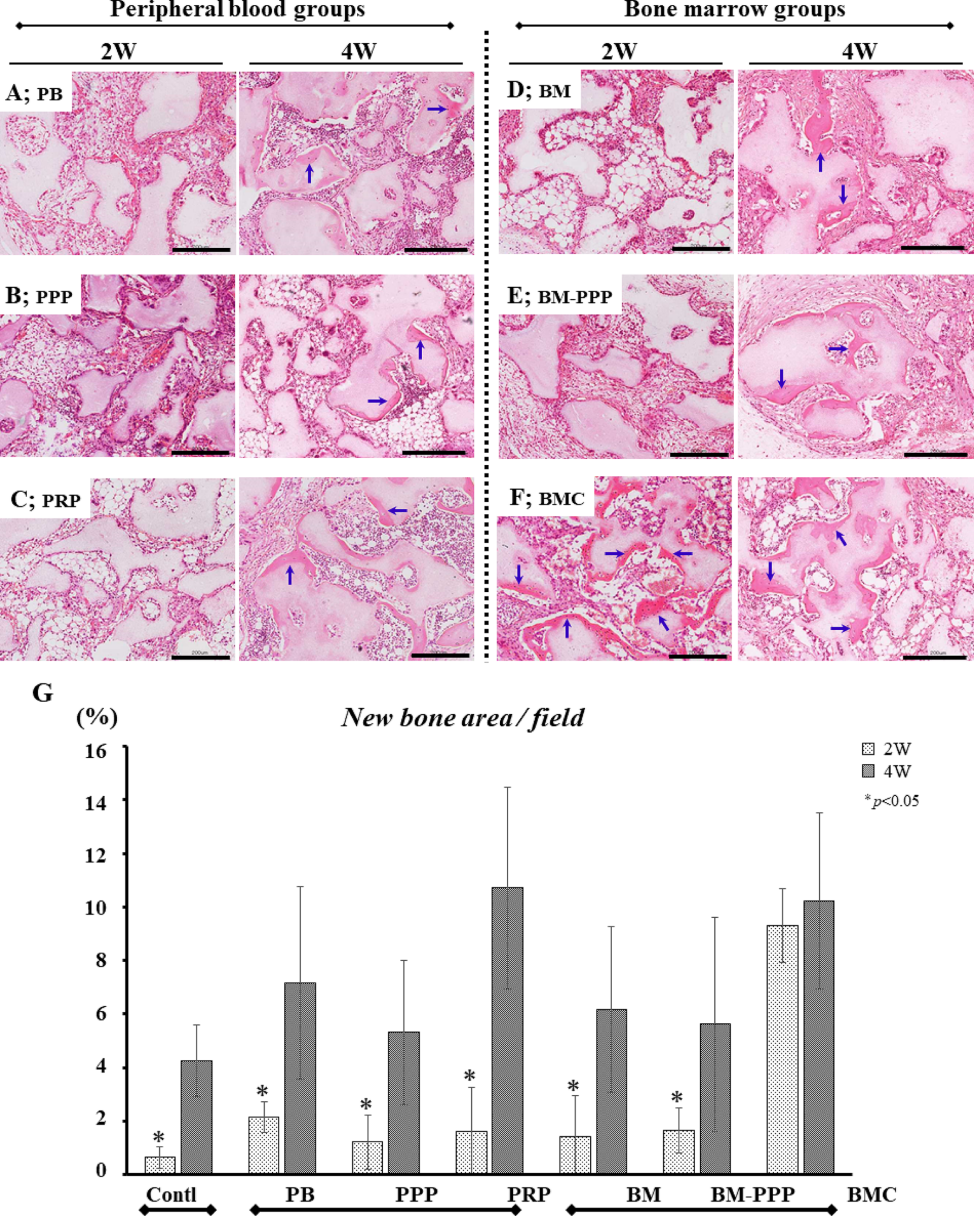
Typical histological appearance in specimens at 2 and 4 weeks following subcutaneous transplantation to the dorsal skin. For each group, sections were stained with hematoxylin and eosin (H&E), and the scale bars represent 200 μm. The left and right panels show the typical appearance at 2 and 4 weeks, respectively, following transplantation. In the **(A)** PB, **(B)** PPP, **(C)** PRP, **(D)** BM, and **(E)** BM-PPP groups, minimal ectopic bone was detectable at 2 weeks but promoted bone formation (blue-arrows) was observed at 4 weeks. In the **(F)** BMC group, abundant ectopic bone (blue-arrows) was observed at 2 and 4 weeks. **(G)** The average area (%) of ectopic bone per whole area was measured in each group. At 2 weeks, the BMC group exhibited significantly bone formation (9.3±1.4%) compared to the other groups (approximately 1–2%) (p<0.05). At 4 weeks, the BMC and PRP groups showed abundant ectopic bone (10.2±3.3% and 10.7±3.8%), however, there were no significant differences among the groups. Values are the means ± standard deviation of five sections from each of the five specimens per group. The asterisk represents statistical significance (*p <0.05) between the BMC group and other groups.

At 2 weeks following transplantation, the lowest level of bone formation was observed in the PB, PPP, PRP, BM, and BM-PPP groups (**Fig 2A–2E**), while abundant ectopic bone was detected in the BMC group (**Fig 2F**). Further, the area of newly formed bone in the BMC group was 9.3±1.4% of the whole area, whereas the other groups showed minimal bone area (approximately 1–2%), levels similar to that observed with a suboptimal-dose of rhBMP-2 (Control group) (**Fig 2G**). However, at 4 weeks post-transplantation, ectopic bone formation was found abundantly in all groups (**Fig 2A–2F**), and the newly formed bone area increased markedly in all groups (**Fig 2G**). In particular, areas in the BMC (10.2±3.3%) and PRP groups (10.7±3.8%) were greater than in the other groups, however, differences among individual groups did not reach statistical significance.

### Histological observations of samples transplanted to the cranium

**Fig 3:**
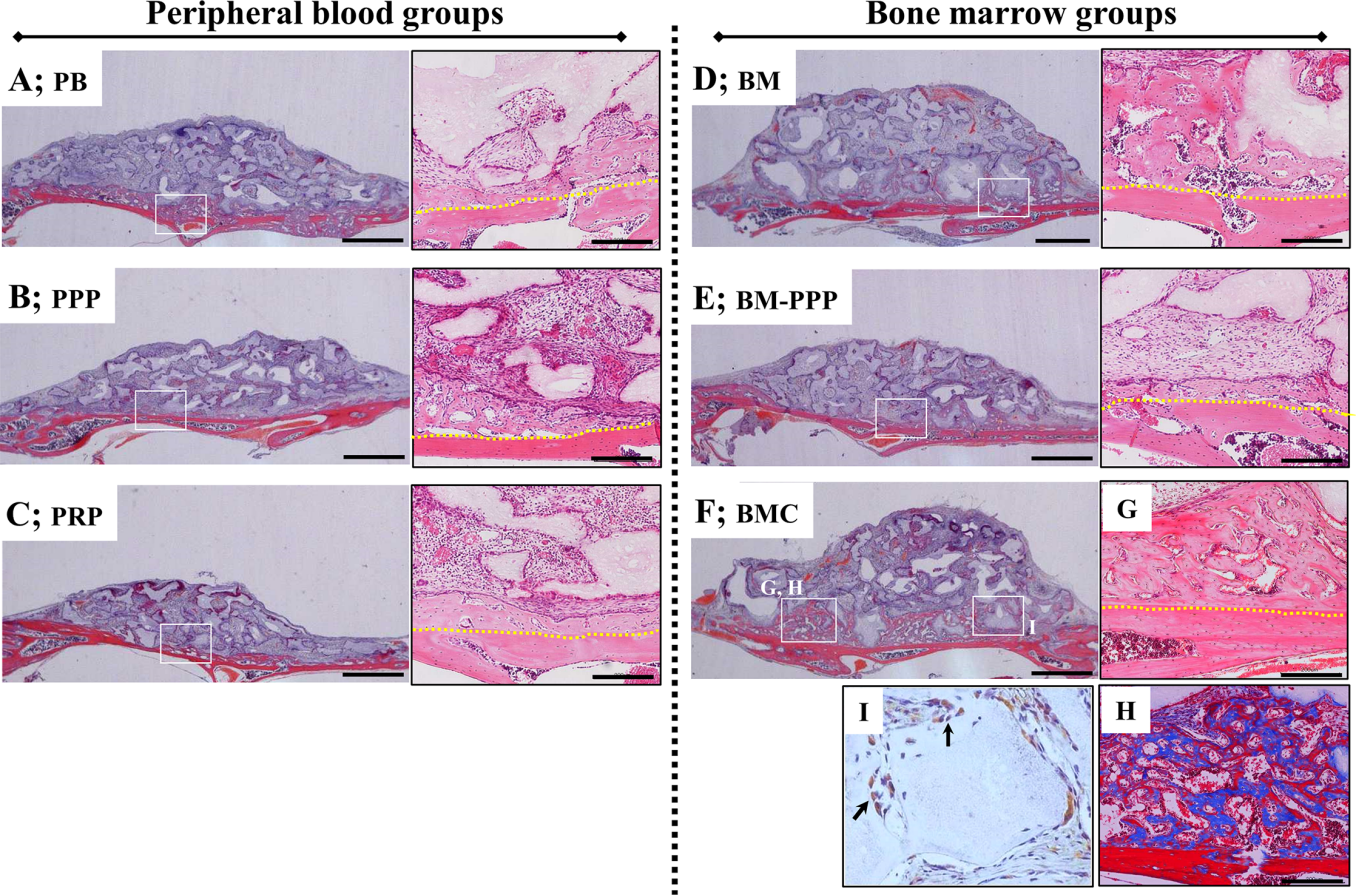
Typical histological appearance in specimens at 2 weeks following transplantation onto the cranium. Coronal plane sections were stained with hematoxylin and eosin (H&E). For each group, the left panel shows the whole area of specimens (scale bars represent 1 mm), and the right panel shows the magnified image of the white box in the left panel (scale bars represent 200 μm). A small amount of new bone was detected along the host bone in the **(A)** PB, (**B**) PPP, **(C)** PRP, and **(E)** BM-PPP groups. The (**D**) BM and (**F**) BMC groups showed obvious bone formation along the host bone. **(G)** New bone area surrounded the β-TCP particles and connected with the host bone [box area in (F)]. **(H)** Masson’s trichrome staining showed the immature (blue) and mature (red) bone in the new bone area [box area in (F)]. (**I**) Human vimentin immuno-staining showed a few positive cells at the surface of newly formed bone adjacent to the β-TCP particles. The yellow dotted line indicates the boundary between the host bone and the specimen.

At 2 weeks following on-lay placement of transplants to the cranium (as an augmentation model), a small amount of new bone was observed in the immediate proximity of the host bone in the PB, PPP, PRP, and BM-PPP groups (**Fig 3A–3C and 3E**). However, considerable bone augmentation was found along the host bone in the BM and BMC groups (**Fig 3D and 3F**), and the new bone was observed to surround the β-TCP particles and connect to the host bone (**Fig 3G–3I**). Samples stained with Masson’s trichrome revealed abundant immature bone (blue-area) in the new bone area connected to the host bone (red area) (**Fig 3G and 3H**). In tracking donor-derived cells stained by human vimentin-antibody in these regions, positive cells could be detected around the surface of newly formed bone in the BMC group (**Fig 3I**).

**Fig 4:**
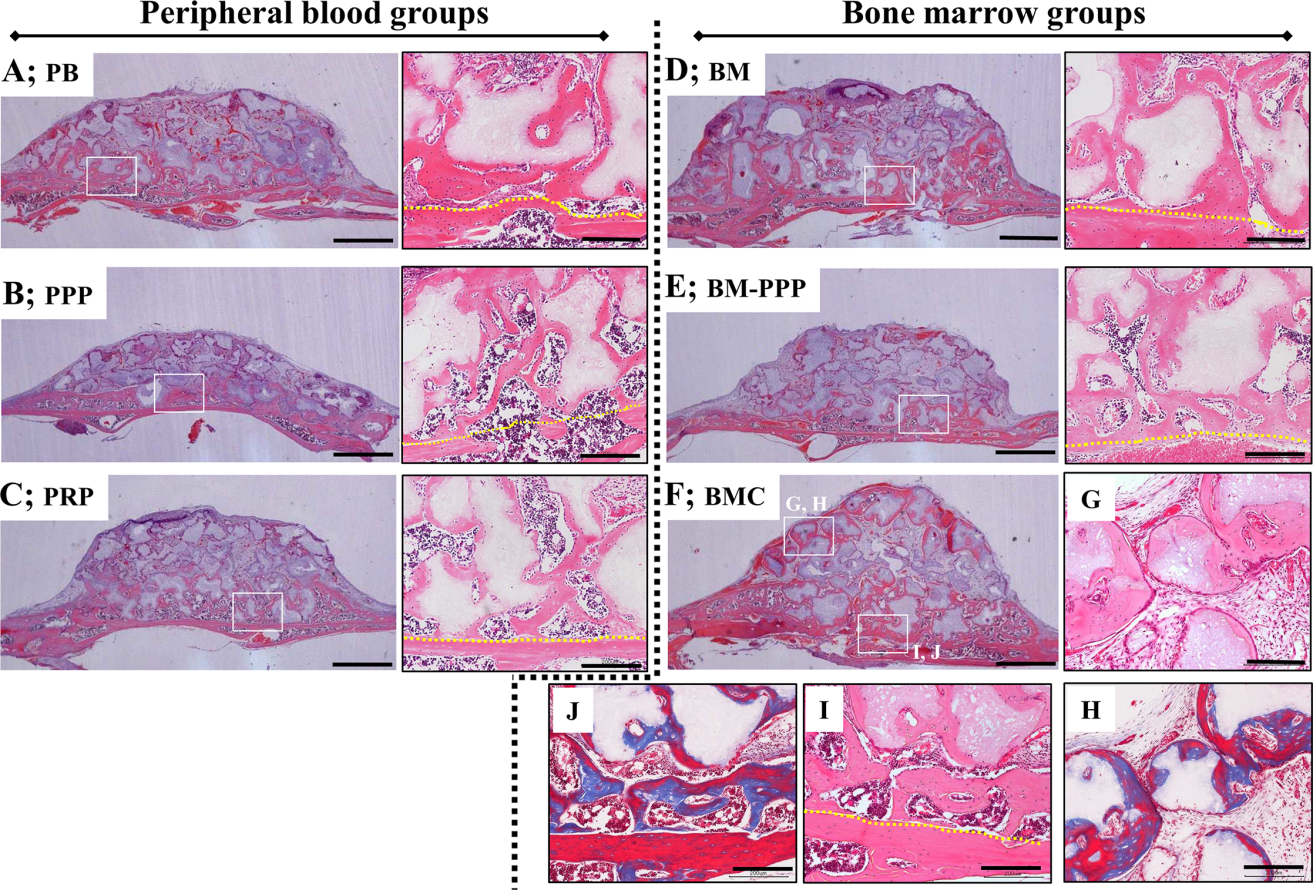
Typical histological appearance in specimens at 4 weeks following transplantation onto the cranium. Coronal plane sections were stained with hematoxylin and eosin (H&E). For each group, the left panel shows the whole area of specimens (scale bars represent 1 mm), and the right panel shows the magnified image of the white box in the left panel (scale bars represent 200 μm). New bone area was obviously promoted along the host bone and β-TCP particles in the **(A)** PB, (**B**) PPP, **(C)** PRP, **(D)** BM, **(E)** BM-PPP, and **(F)** BMC groups, and replacement bone tissue was clearly observed at the surface of the β-TCP particles in the magnified areas. **(G, H)** The surface of β-TCP particles was being resorbed and replaced with new bone at the far site from the host bone, and newly formed bone was observed to be more mature (red stained by Masson’s trichrome) [box area in (F)]. **(I, J)** The newly formed bone was sufficiently integrated with the host bone, and appeared mature (red stained by Masson’s trichrome) [box area in (F)]. The yellow dotted line indicates the boundary between the host bone and the specimen.

At 4 weeks following transplantation, newly formed bone was clearly observed along the host bone and β-TCP particles in all groups, and most of this bone connected to the host bone (**Fig 4A–4F**). Furthermore, replacement of the bone tissues, which included osteocytes, was detected at the surface of the absorbed TCP granules (**Fig 4A–4G and 4I**). However, we found the new bone area was markedly augmented in the BMC group (**Fig 4F**). Specifically, replacement of the newly formed bone at the surface of β-TCP granules had progressed further towards sites far from the host bone (**Fig 4G**), and the augmented bone appeared to be mature (red area stained by Masson’s trichrome) (**Fig 4H and 4J**).

**Fig 5:**
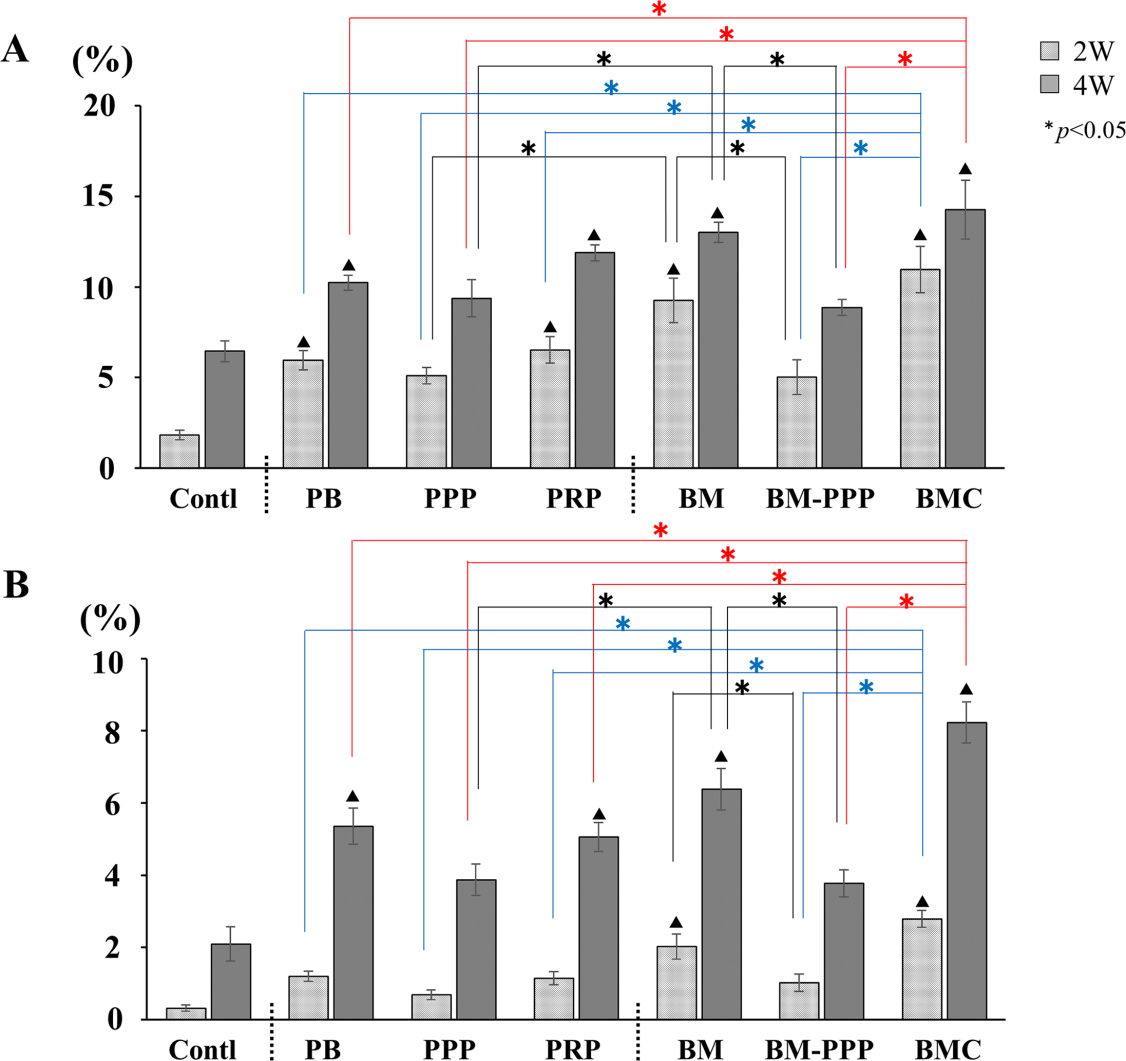
**Average area (%) of newly formed bone in the whole area (A) and at the surface of β-TCP particles (B). (A**) At 2 weeks, BMC and BM groups showed considerable new bone formation, and the amount of new bone in the BM group was significantly increased compared to the Control, PB, PPP, PRP, and BM-PPP groups (p <0.05). At 4 weeks, bone formation was promoted in all groups. In particular, new bone area was obviously augmented in the BMC, BM, and PRP groups. **(B)** When the area of replaced bone tissue at the surface of β-TCP particles was assessed, BMC and BM groups showed significantly increased area compared to the Control group at 2 weeks (p <0.05). Then, the BMC group showed more prominent bone formation at 4 weeks. In particular, a significant difference was found between BMC and PRP groups. Values are the means ± standard deviations of five sections from each of the five specimens per group. The asterisk represents statistical significance (*p <0.05) among experimental groups, and the triangle-mark represents statistical significance (^▲^p <0.05) between the Control and other groups.

Areas of augmented bone tissues were compared between groups. At 2 weeks, the areas of newly formed bone in the PB, PRP, BM, and BMC groups increased significantly compared with the Control (**Fig 5A**). Particularly, the BM and BMC groups showed greater area of bone formation. Moreover, we found the area replaced by new bone at the surface of the β-TCP granules was significantly augmented in the BM and BMC groups 2 weeks post-transplantation (**Fig 5B**). At 4 weeks, although bone formation was promoted in all groups, the PRP, BM and BMC groups exhibited greater bone growth compared to the other groups (**Fig 5A**). Meanwhile, replacement by new bone at the surface of the absorbed β-TCP granules was significantly promoted compared with the other groups, particularly the PRP group (**Fig 5B**).

### Synergistic effect on bone augmentation

**Fig 6:**
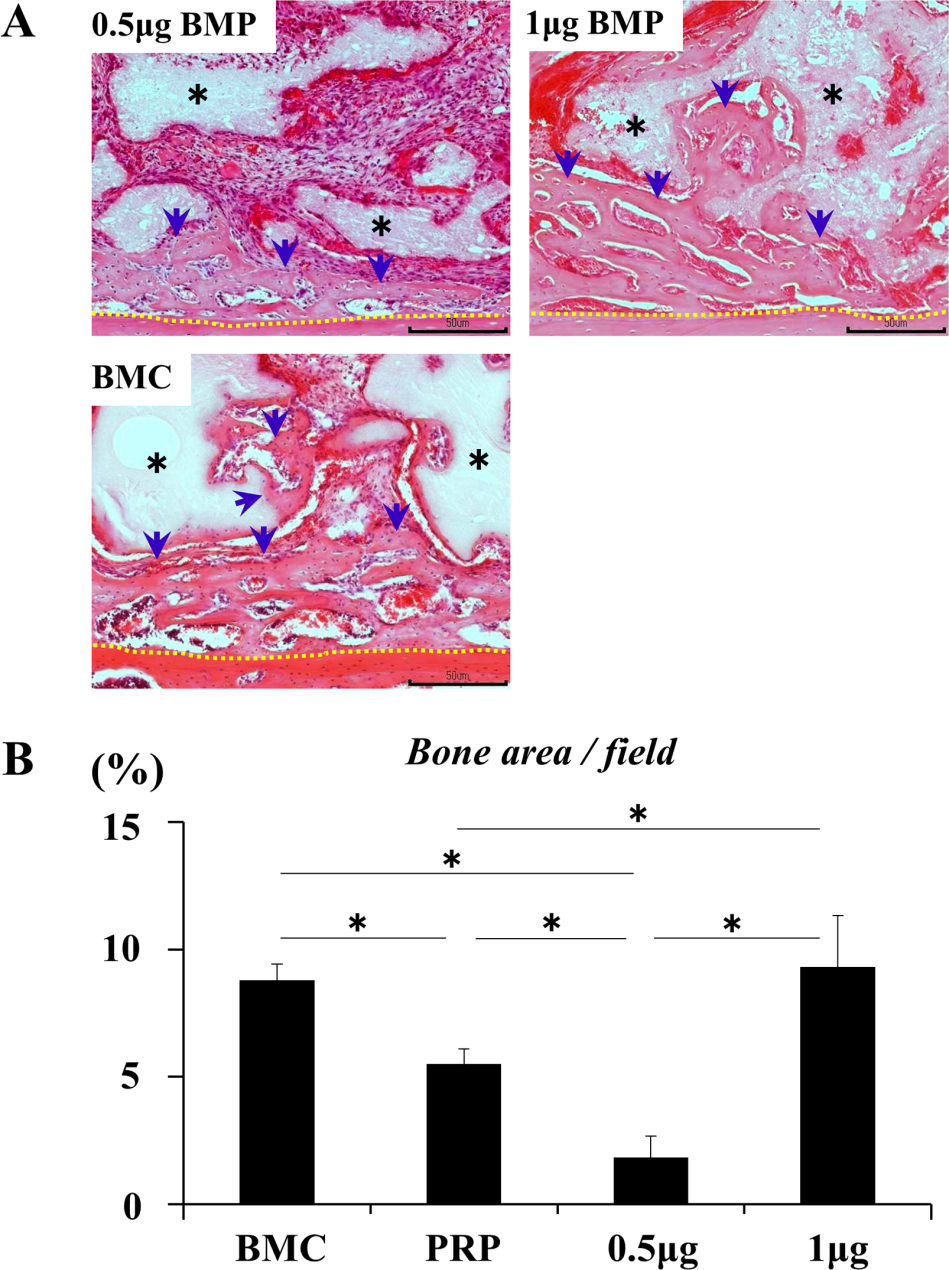
Synergistic effect of BMC and BMP-2 on bone formation. **(A)** Bone formation at 2 weeks post-transplantation. Scale bars represent 200 μm. The newly formed bone was integrated with the host bone. rhBMP-2 at 0.5 μg induced a limited amount of new bone formation while 1 μg rhBMP-2 and BMC (BMC represents the BMC group) promoted bone formation to surround β-TCP particles and connect to the host bone. Black asterisk: β-TCP particles, blue arrow: newly formed bone, and yellow dotted line: boundary between the host bone and specimen. **(B)** Comparison of the area (%) of newly formed bone in each group. PRP represents the PRP group. Values are the means ± standard deviations of five sections from each of the five specimens per group. The asterisk represents statistical significance (*p <0.05) among groups.

Bone formation at 2 weeks was promoted synergistically by a suboptimal-dose (0.5 μg) of rhBMP-2 and BMC to the same level as observed for the optimal-dose (1 μg) of rhBMP2 (**Fig 6A and 6B**). This synergistic effect was greater than the effects of a suboptimal-dose of rhBMP-2 and PRP individually (**Fig 6B**).

## Discussion

This study demonstrated the utility of co-delivery of BMC and a suboptimal (low) dose of rhBMP2 to regenerate bone tissue. The study outcomes were: 1) BMC accelerated bone formation from the early-phase of transplantation when co-delivered with a suboptimal-dose of rhBMP-2, 2) BMC rather than PRP enabled a reduction in the dose of rhBMP-2 by its synergistic action *in vivo*. These outcomes suggest that promotion of this synergism might have arisen from the response of concentrated MNCs to rhBMP-2.

With respect to *in vivo* osteoinduction, we found that BMC could markedly hasten ectopic bone formation in subcutaneous tissue. The amount of newly formed bone at 2 weeks in the BMC group was 9.3±1.4% while that in the PRP group was 1.6±1.7%. This phenomenon might result from the interaction between bone marrow MNCs and the suboptimal-dose of rhBMP-2. In this study, we concentrated MNCs in BMC to approximately 2.5-fold that of PRP or BM. However, at 4 weeks bone formation was obviously promoted in the PRP group to the same level (approximately 10%) as the BMC group while the BM group did not show similar levels of bone formation. This result strongly suggested that growth factors released from platelets in the PRP function to promote bone formation in the presence of rhBMP-2, as evidenced by the low numbers of platelets in BM compared to PRP. Although the exact effect of PRP on osteogenesis remains controversial, several studies have shown that PRP does not positively affect early stage bone regeneration [34,35]. However, in our previous study, we found that remarkable bone formation from an early stage was induced in the presence of both PRP and an optimal dose of rhBMP-2 [16]. Therefore, as with the PRP group in this study, we speculate that the reduction in rhBMP-2 concentration to a suboptimal-dose led to a delay in osteogenesis. rhBMP-2 likely affected the increased osteoprogenitor cells recruited by growth factors contained in the PRP group [16,36]. In fact, it has been shown that the co-delivery of SDF-1 and a suboptimal-dose of rhBMP-2 promoted osteoinduction to comparable levels seen with the optimal-dose of rhBMP-2 in rat calvarial bone defects [15]. SDF-1 is one of several growth factors contained in PRP [37], and is known to facilitate wound healing through augmented recruitment of bone marrow MSCs or EPCs to injured sites. However, besides SDF-1, PRP releases several other growth factors such as PDGF, TGF-β, and VEGF [38–40]. Therefore, the interaction between osteoprogenitor cells, which are recruited by several growth factors released from PRP, and a suboptimal-dose of rhBMP-2 may obviously induce ectopic bone formation at 2 weeks and later. In contrast, concentrated MNCs in BMC and a suboptimal-dose of rhBMP-2 might be able to interact with each other directly at the early stage of transplantation. Indeed, in this study, we found that BMC-derived mesenchymal cells survived along the surface of new bone formation on the cranium at 2 weeks post-transplantation, leading to the assumption that bone marrow MNCs can participate in bone regeneration at the early stage of transplantation. This assumption may be supported by the result of a previous clinical study on maxillary sinus augmentation which revealed that BMC induces quicker bone formation with bone mineral substitutes [41].

A similar general trend was observed when specimens were transplanted to the mouse cranium as a bone augmentation model. This model can be considered appropriate for alveolar ridge augmentation. However, in this model, BMC was superior to PRP in inducing bone augmentation. Indeed, from 2 to 4 weeks post-transplantation, the amount of augmented bone was enhanced from approximately 11 to 14% in the BMC group, while the PRP group showed augmentation from approximately 6 to 11%. Moreover, interestingly, even the BM group promoted bone formation, from approximately 9 to 13% of the whole area. These findings indicate that bone marrow MNCs, and not platelets or peripheral blood MNCs, affect bone formation in the presence of rhBMP-2. At present, comparative studies regarding the performance of BMC and PRP in bone regeneration are scarce [42,43], but we have previously demonstrated that BMC and PRP possess a similar ability to accelerate bone augmentation when transplanted onto the mouse calvarium without rhBMP-2 [21]. At that time, we concluded that PRP is a more realistic clinical option for bone engineering. However, in the presence of a suboptimal-dose of rhBMP-2, there are discrepancies in the amounts of augmented bone between the BMC and PRP groups and thus BMC may be highly useful in clinical applications of rhBMP-2 for bone engineering. Although the actual clinical significance of BMC remains unclear, it may reduce a certain amount of rhBMP-2 without major loss of functionality. In this study, we found that in the presence of BMC, a suboptimal-dose (0.5 μg) of rhBMP-2 induced bone augmentation on the cranium to the same level as the optimal-dose (1.0 μg), suggesting that BMC can reduce the amount of rhBMP-2 by one-half. A previous clinical study demonstrated that the adverse effects of rhBMP2 treatment could be eliminated by reducing the optimal-dose (1.5 mg/ml) by one-half (0.75 mg/ml) when transplanted to the maxillary sinus floor [44]. Therefore, co-delivery of BMC appears to rescue the reduced osteoinductive function of a suboptimal-dose rhBMP-2, and it may help attenuate the adverse effects of rhBMP-2 treatment. However, such combination treatments may cause unexpected adverse events and there are several study limitations to this investigation. This study employed immunodeficient-mice and their immunoreaction to rhBMP-2 must be largely inhibited. Indeed, even the optimal-dose (1.0 μg) in this study (approximately 0.05 μg/g body weight) was lower than the clinical suboptimal-dose (0.75 mg/ml) for patients (approximately 0.1 μg/g body weight). Therefore, although the described combination treatment with BMC and a suboptimal-dose of rhBMP-2 may demonstrate superior efficacy for bone formation, further investigations aimed at understanding potential side effects are required to determine the most appropriate and safe use of rhBMP-2 in humans [45].

In conclusion, our findings revealed that both BMC and PRP enhance the osteoinductive function of a suboptimal-dose of rhBMP2 synergistically. However, co-delivery of BMC may be a more direct approach to promote bone regeneration while avoiding undesirable side effects. Although the detailed mechanisms that lead to the promotion of bone regeneration remain unclear, the enhanced osteoinducibility of the alloplastic substitutes by the synergistic effect of BMC and low-dose rhBMP2 is a potential highly relevant approach to clinical bone reconstruction in the near future. Utilization of BMC may bypass the time consuming and technically difficult process of cell expansion and differentiation, enabling both harvesting and transplanting of BMC during the same surgical procedure [46]. Therefore, although there are study limitations, this investigation suggests that BMC transplantation facilitates the clinical use of rhBMP-2 as an alternative strategy for bone engineering.

## Supporting information

S1 Table Concentration rates in each fraction. The upper panel represents the concentration rate of mononuclear cells (MNCs) and the lower panel represents the concentration rate of platelets (PLTs) in PRP, PPP, BMC and BM-PPP groups.
(XLS)

S2 Table Raw data after measurements of newly formed bone area and percentages of bone area in the whole area on the bone augmentation model. (XLSX)
